# Laparoscopy in abdominal trauma at a high-volume Level 1 trauma centre in a low- and middle-income setting: A 54-month retrospective analysis

**DOI:** 10.4102/jcmsa.v4i1.494

**Published:** 2026-10-28

**Authors:** Lebone Mohlala, Maeyane S. Moeng, Ekene E. Nweke, Shumani Makhadi

**Affiliations:** 1 Department of Surgery, School of Clinical Medicine, Faculty of Health Sciences, University of the Witwatersrand, Johannesburg, South Africa

**Keywords:** laparoscopy, penetrating abdominal trauma, low- and middle-income countries, trauma surgery, minimally invasive surgery, South Africa

## Abstract

**Background:**

Laparoscopy is a valuable surgical modality in trauma, yet its application remains underexplored. This study evaluates the feasibility and short-term outcomes of laparoscopy for abdominal trauma at a high-volume Level 1 trauma centre in Johannesburg.

**Methods:**

A retrospective cohort study of adult patients who underwent laparoscopy for abdominal trauma at Charlotte Maxeke Johannesburg Academic Hospital from January 2021 to June 2025 was conducted. Demographics, mechanism of injury, injury severity score, intraoperative findings, conversion rates, complications and length of stay were analysed.

**Results:**

Of 69 patients who underwent laparoscopy, 60 met the inclusion criteria. Median age was 30.5 years (IQR 27–38), with 95% male patients (95% CI: 86.3%–98.3%). Penetrating trauma accounted for 98.3% of cases. Negative laparoscopy occurred in 28.3% (95% CI: 18.5%–40.8%), potentially avoiding non-therapeutic laparotomy. Diaphragmatic injuries were the most common positive finding (15%). Conversion occurred in 23.3% (95% CI: 14.4%–35.4%), most commonly for definitive repair (15%). Complications occurred in 6.7% (95% CI: 2.6%–15.9%), with no in-hospital mortalities. Median length of stay was 4 days (IQR 3–7).

**Conclusion:**

Laparoscopy is feasible in carefully selected haemodynamically stable trauma patients, even in resource-constrained, high-volume settings. These findings support further investigation of laparoscopy in low- and middle-income countries, with investment in training infrastructure a key priority.

**Contribution:**

This study provides contemporary South African evidence on the feasibility and short-term outcomes of laparoscopy in selected trauma patients and supports further evaluation of its potential to avoid non-therapeutic laparotomy.

## Introduction

Trauma remains one of the most pressing global health challenges, responsible for approximately 4.4 million deaths annually and disproportionately affecting low- and middle-income countries (LMICs), where over 90% of injury-related mortality occurs.^1^ South Africa epitomises this crisis: the country’s trauma rates are estimated at five to nine times the global average, driven by a complex interplay of poverty, inequality, substance misuse and interpersonal violence.^1,2^ This burden falls heavily on an already strained public health system, where emergency surgical volumes vastly exceed elective caseloads, and where the emergency-to-elective surgical ratio can exceed 145:1 for abdominal procedures.^3^

The management of penetrating abdominal trauma has evolved substantially since laparotomy became the standard of care during World War I.^4^ While mandatory exploration was historically the default approach, it carried a significant price: non-therapeutic laparotomy rates of up to 61% in some series,^5^ exposing patients to avoidable morbidity, including a 14.5% complication rate, prolonged hospital stays, increased risk of surgical site infections, adhesive bowel obstruction and paradoxically, even increased mortality.^6,7^ These consequences are amplified in resource-limited settings where hospital beds are scarce, theatre access is competitive and every unnecessary surgical day represents a lost opportunity for another patient awaiting care.

Laparoscopy offers a compelling alternative. In elective surgery, it has consistently demonstrated advantages including reduced postoperative pain, shorter hospital stays, decreased wound complications and fewer adhesions.^8^ Its diagnostic accuracy approaches 100% for peritoneal penetration and diaphragmatic injuries, while simultaneously offering the opportunity for therapeutic intervention.^5,9^ Yet despite these advantages, the adoption of laparoscopy in trauma, particularly in LMICs, has been slow. Systematic reviews have identified critical barriers including equipment costs, limited training infrastructure, scarcity of experienced mentors and inconsistent access to basic resources such as reliable electricity and sterile supplies.^10,11^ In sub-Saharan Africa, where the surgical workforce is already insufficient and overwhelmed by an extraordinary trauma burden, these barriers are particularly acute.

Emerging evidence from centres in South Africa, Egypt, Brazil and Colombia has begun to demonstrate the feasibility of laparoscopy in trauma.^5,8,9,12,13,14^ A previous study from our institution demonstrated the safe introduction of laparoscopy in 54 trauma patients, with a conversion rate of 14.8% and no mortality.^12^ However, the evidence base remains limited, with most series comprising fewer than 60 patients, and virtually no data on the sustained use of this modality in high-volume LMIC trauma centres over extended periods.

This study builds upon our institution’s prior work and represents one of the larger single-centre series of trauma laparoscopy from a Level 1 trauma centre operating under significant resource constraints in Johannesburg, South Africa. We aimed to evaluate whether laparoscopy remains feasible when applied as a sustained practice over a 54-month period in a setting where high patient volumes compete with limited theatre access and evolving surgical expertise.

The primary aim of this study was to evaluate the feasibility and describe the short-term outcomes of laparoscopy for managing abdominal trauma in a selected cohort of haemodynamically stable patients at a high-volume, resource-constrained Level 1 trauma centre in Johannesburg, South Africa. Secondary aims were to describe conversion rates and their indications, characterise complications and re-operation rates, and compare our outcomes with published series from similar settings.

## Research methods and design

### Study design and setting

This was a retrospective cohort study of all adult patients (≥ 18 years) who underwent laparoscopy for abdominal or thoracoabdominal trauma at Charlotte Maxeke Johannesburg Academic Hospital (CMJAH) over a 54-month period from 01 January 2021 to 30 June 2025. This study was conducted and reported in accordance with the Strengthening the Reporting of Observational Studies in Epidemiology (STROBE) guidelines. Charlotte Maxeke Johannesburg Academic Hospital is a 1088-bed tertiary academic hospital and designated Level 1 trauma centre affiliated with the University of the Witwatersrand, serving a large urban catchment area in Gauteng province, South Africa. The hospital manages a high volume of penetrating and blunt trauma, reflecting the significant burden of violence-related injury in the region.

### Patient selection

From the hospital trauma registry, a total of 69 patients who underwent laparoscopy during the study period were identified using the departmental MediBank database, REDCap electronic data capture system and hospital records. Nine patients were excluded because of incomplete medical records. The primary analysis cohort comprised 60 patients with complete records. A total of 1340 laparotomies were performed over the study period. Patient selection for laparoscopy followed institutional criteria developed over the prior institutional series. Laparoscopy was considered for haemodynamically stable patients (systolic blood pressure > 90 mmHg, without requirement for vasopressor support) with clinical or imaging evidence of abdominal injury requiring operative assessment. Specific indications included: (1) bowel or omental evisceration; (2) clinical or cross-sectional imaging findings suggesting visceral injury (e.g. pneumoperitoneum or free fluid on computed tomography [CT]); (3) suspected diaphragmatic injury, particularly following thoracoabdominal stab wounds; and (4) clinical signs of peritonitis in a haemodynamically stable patient. Contraindications to laparoscopy included haemodynamic instability despite resuscitation, massive haemoperitoneum on imaging or significant coagulopathy. The decision between laparoscopy, open laparotomy and non-operative management was made by the attending trauma surgeon based on clinical assessment, admission physiology and available imaging. Blunt trauma patients were considered for laparoscopy if haemodynamically stable with CT evidence of intra-abdominal injury requiring operative clarification.

### Data collection

Variables collected included patient demographics (age, sex), mechanism of injury, Injury Severity Score (ISS), admission clinical parameters (systolic blood pressure, arterial pH, base excess, and lactate), intraoperative findings, surgical intervention performed, conversion to open surgery, complications, re-operation and length of hospital stay.

Outcome definitions: Negative laparoscopy was defined as laparoscopic exploration revealing no intra-abdominal injury requiring intervention. Therapeutic laparoscopy was defined as laparoscopy during which a definitive operative procedure was performed (repair, resection or haemostasis). Conversion was defined as the decision to proceed to open laparotomy after commencement of laparoscopy, regardless of reason. Iatrogenic injury was defined as an organ or vessel injury occurring as a direct consequence of the operative procedure. Missed injury was defined as an injury not identified at the index operation and requiring re-operation within the same hospital admission. Complication was defined as any unplanned deviation from the expected postoperative course requiring further intervention or extending hospital stay. Re-operation was defined as a return to theatre within the index admission. Mortality was defined as in-hospital death during the index admission. The assessment window for all outcomes was the index hospital admission.

### Statistical analysis

All statistical analyses were performed using Stata version 19 (StataCorp, College Station, TX, United States). Continuous variables were assessed for normality using the Shapiro–Wilk test. As continuous variables demonstrated non-normal distributions, they are reported as medians with interquartile ranges (IQR). Categorical variables are presented as frequencies and percentages with 95% confidence intervals (CI) calculated using the Wilson score method. Spearman’s rank correlation coefficient was used to evaluate associations between continuous clinical variables and length of hospital stay. A *p*-value of < 0.05 was considered statistically significant.

### Ethical considerations

Ethics approval was obtained from the University of the Witwatersrand Human Research Ethics Committee (Ref: M2507110), and institutional permission was granted by the Chief Executive Officer of Charlotte Maxeke Johannesburg Academic Hospital. The study was registered on the National Health Research Database.

## Results

### Patient demographics and injury profile

Sixty patients met the inclusion criteria during the 54-month study period (January 2021 to June 2025). A total of 1340 laparotomies were performed over the study period; 60 patients who met the criteria were included in this analysis. The cohort was predominantly male (*n* = 57, 95.0%, 95% CI: 86.3% – 98.3%), with a median age of 30.5 years (IQR 27.0–38.0).

Penetrating trauma accounted for most cases (*n* = 59, 98.3%), with thoracoabdominal stab wounds being the most common mechanism (*n* = 28, 46.7%), followed by multiple stab wounds, including the abdomen (*n* = 9, 15.0%), abdominal stab wounds (*n* = 7, 11.7%) and abdominal gunshot wounds (*n* = 7, 11.7%). Only one patient (1.7%) underwent laparoscopy for blunt abdominal trauma for assessment of free fluid in the abdomen secondary to pelvic fractures. The distribution of injury mechanisms is presented in Table 1.

**TABLE 1:** Demographic and injury characteristics of the study cohort (*N* = 60).

Characteristic	Category	*n*	%	Median	IQR
Sex	Male	57	95.0	-	-
	Female	3	5.0	-	-
Age (years)	-	-	-	30.5	27.0–38.0
Mechanism of injury	Thoracoabdominal stab	28	46.7	-	-
	Multiple stab wounds	9	15.0	-	-
	Abdominal stab	7	11.7	-	-
	Abdominal gunshot	7	11.7	-	-
	Thoracoabdominal gunshot	4	6.7	-	-
	Pelvic gunshot	4	6.7	-	-
	Blunt abdominal trauma	1	1.7	-	-
ISS (*n* = 60)	1–4 (minor)	36	61.0	-	-
	5–9 (moderate)	15	25.4	-	-
	10–12 (moderate–severe)	6	10.0	-	-
	13–17 (severe)	3	5.1	-	-

IQR, interquartile range; ISS, injury severity score.

### Admission clinical parameters

All patients were haemodynamically stable at the time of operative decision-making. The median systolic blood pressure on arrival was 129.5 mmHg (IQR 115.5–139.5). Arterial blood gas parameters demonstrated a median pH of 7.366 (IQR 7.334–7.391), median base excess of −3.55 (IQR −5.75 to −2.05) and median lactate of 2.1 mmol/L (IQR 1.35–3.75). These parameters are consistent with haemodynamic stability and informed the clinical decision to proceed with laparoscopy in accordance with selection criteria (Table 2).

**TABLE 2:** Admission clinical parameters (*N* = 60).

Parameter	Median	IQR	Median	IQR
Systolic BP (mmHg)	131.2	22.4	129.5	115.5–139.5
pH	7.364	0.055	7.366	7.334–7.391
Base excess (mmol/L)	−3.49	3.21	−3.55	−5.75 to -2.05
Lactate (mmol/L)	2.6	1.77	2.1	1.35–3.75

BP, blood pressure; IQR, interquartile range.

### Intraoperative findings and intervention

In all, 24 patients (40.0%) required no therapeutic procedure. Of note, 17 of these patients had truly negative laparoscopy (no identifiable injury), while the remaining seven had positive findings (including minor haematomas or haemoperitoneum) identified at laparoscopy that were managed non-operatively. Among patients with positive findings, diaphragmatic injuries were the most identified (*n* = 9, 15.0%), followed by solid organ injuries (*n* = 4, 6.7%), small bowel injuries (*n* = 2, 3.3%), colon injuries (*n* = 2, 3.3%) and a single gastric injury (1.7%). Twelve patients (20.0%) had multiple combined injuries.

Definitive laparoscopic repair was successfully performed in 19 patients (31.7%, 95% CI: 21.3% – 44.2%), including primary repair of diaphragmatic and hollow visceral injuries (Figure 1). Drain insertion was performed in three patients (5.0%) and abdominal washout in one (1.7%). Fourteen patients (23.3%, 95% CI: 14.4% – 35.4%) required conversion to open laparotomy: nine (15.0%) for definitive repair that could not be accomplished laparoscopically, four (6.7%) for exploration of retroperitoneal haematomas and one (1.7%) for poor visualisation (see Table 3).

**FIGURE 1:**
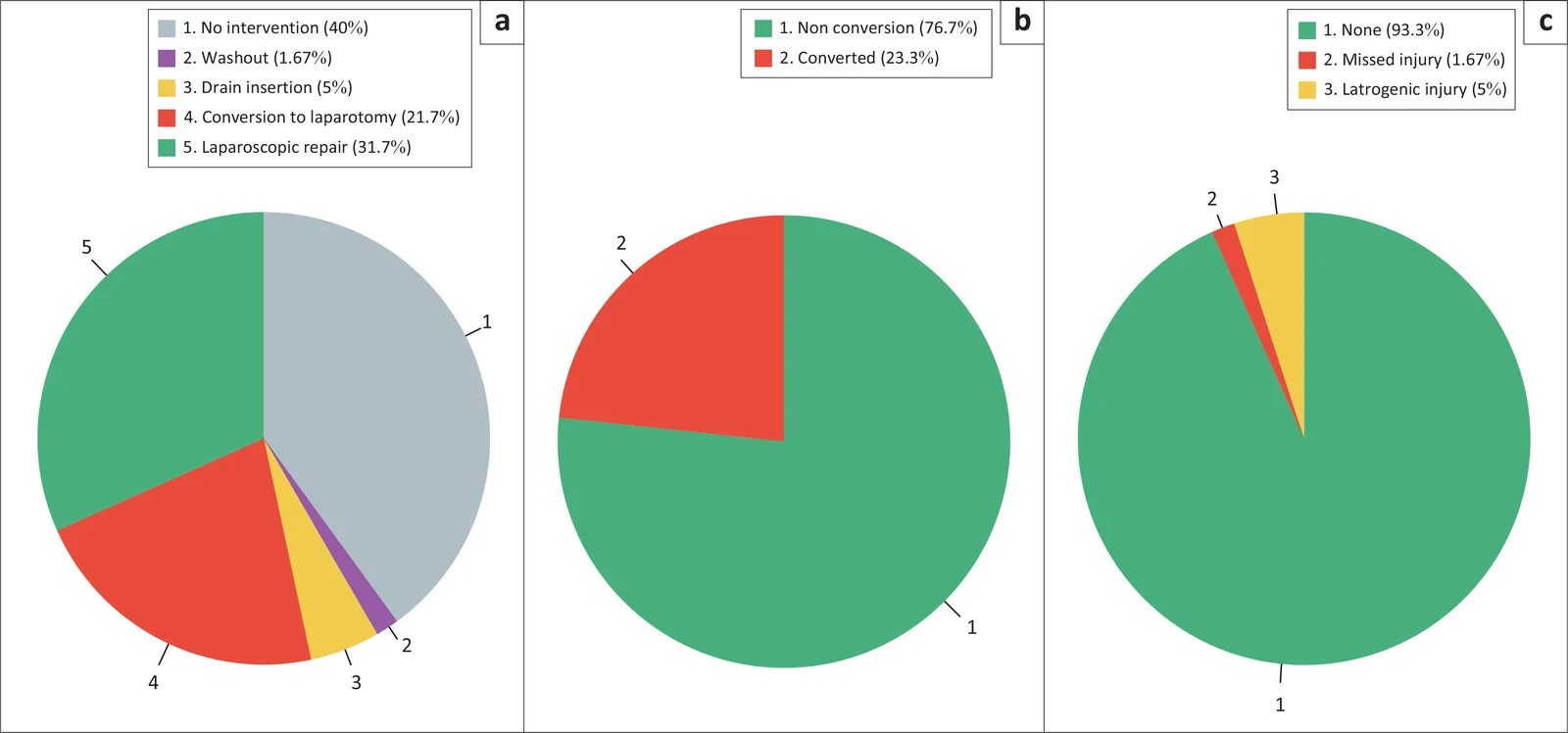
Surgical outcomes overview showing (a) intervention type, (b) conversion and (c) complications (*n* = 60).

**TABLE 3:** Intraoperative findings, intervention and outcomes (*N* = 60).

Parameter	Category	*n*	%
Intraoperative findings	Negative	17	28.3
	Diaphragmatic injury	9	15.0
	Small bowel injury	2	3.3
	Colon injury	2	3.3
	Stomach injury	1	1.7
	Solid organ injury	4	6.7
	Other†	13	21.7
	Multiple injuries combined	12	20.0
Intervention	No intervention	24	40.0
	Laparoscopic repair	19	31.7
	Drain insertion	3	5.0
	Washout	1	1.7
	Conversion to laparotomy	14	23.3
Reason for conversion	Definitive repair	9	15.0
	Exploration of haematoma	4	6.7
	Poor visualisation	1	1.7
Complications	None	56	93.3
	Iatrogenic injury	3	5.0
	Missed injury	1	1.7
Re-operation	None	57	95.0
	Laparotomy	3	5.0

† , Other: abdominal wall injuries, haematoma, adhesions, haemoperitoneum.

### Complications and re-operations

The overall complication rate was 6.7% (*n* = 4, 95% CI: 2.6% – 15.9%) (see Figure 1). Three patients (5.0%) sustained iatrogenic injuries during laparoscopy: two did not require further intervention, while one required splenectomy for an iatrogenic splenic injury with ongoing haemorrhage. One patient (1.7%) had a missed colon injury identified on subsequent clinical assessment requiring re-operation. There were no in-hospital mortalities in this cohort.

Three patients (5.0%, 95% CI: 1.7% – 13.7%) required re-operation, all by open laparotomy: one for a missed colon injury, one for retrieval of a retained suture needle and one for splenectomy following an iatrogenic splenic injury with ongoing haemorrhage.

### Length of stay and clinical correlations

The median length of hospital stay was 4 days (IQR 3–7). The distribution was markedly right-skewed, reflecting a small number of patients with prolonged admissions (Table 4).

**TABLE 4:** Spearman rank correlation between clinical parameters and length of hospital stay (*N* = 60).

Variable	Spearman’s *ρ*	*p*-value
Age	+0.230	0.077
Systolic blood pressure	+0.158	0.228
pH	−0.049	0.712
Base excess	−0.077	0.557
Lactate	−0.121	0.356

Note: No statistically significant correlations were identified at the *p* < 0.05 level.

Spearman rank correlation analysis was performed to assess the association between continuous clinical variables and length of hospital stay. No statistically significant correlations were identified between length of stay and age (*ρ* = 0.230, *p* = 0.077), systolic blood pressure (*ρ* = 0.158, *p* = 0.228), pH (*ρ* = −0.049, *p* = 0.712), base excess (*ρ* = −0.077, *p* = 0.557) or lactate (*ρ* = −0.121, *p* = 0.356) (Table 4).

### Comparison with published literature

Table 5 summarises the findings of this study alongside published series evaluating laparoscopy in abdominal trauma. Our cohort of 60 patients represents one of the larger single-centre series in the LMIC literature. The zero in-hospital mortality rate in our series is consistent with the majority of published studies^5,8,14,15,16,17,18^; two series reported mortality (Koto et al.^13^: 11.4%, and Gao et al.^16^: 1.9%), both predominantly in blunt trauma cohorts (Table 5). Our conversion rate of 23.3% falls within the reported range of 0% – 26.7% across centres (Figure 2).

**FIGURE 2:**
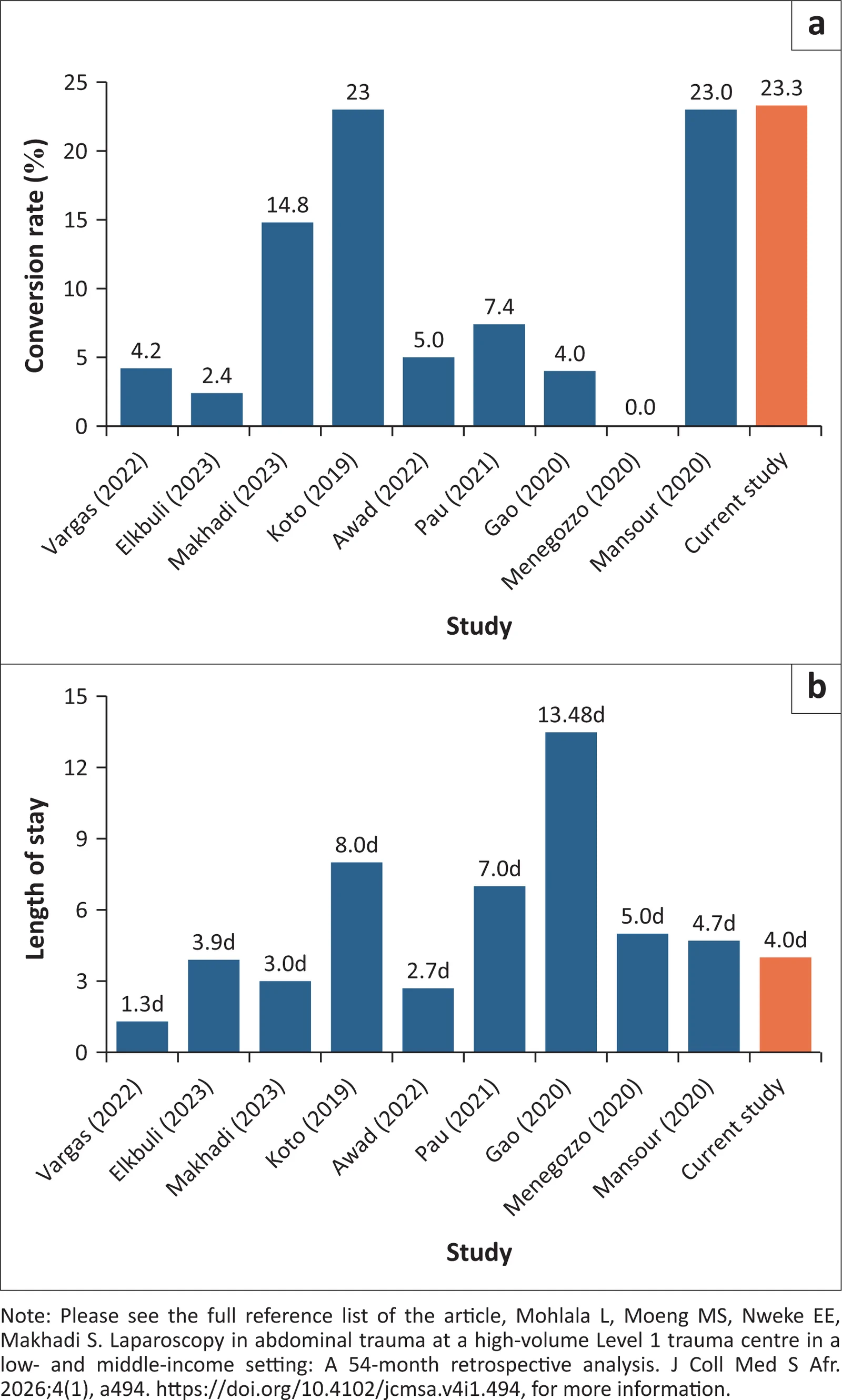
Comparison of conversion rates (a) and length of stay (b) across published laparoscopic trauma series. Current study highlighted in orange.

**TABLE 5:** Comparison of laparoscopic trauma series across centres.

Study	*n*	Setting	Mortality (%)	Negative laparoscopy (%)	Conversion (%)	LOS (days)
Vargas et al.^5^	24	Colombia	0.0	8.3	4.2	1.3
Elkbuli et al.^8^	486	US	1.2	N/A	N/A	3.9
Makhadi et al.^12^	54	South Africa	0.0	16.7	14.8	3.0
Koto et al.^13^	35	South Africa	11.4	34.3	23.0	11.0
Awad et al.^14^	40	Egypt	0.0	10.0	5.0	N/A
Pau et al.^15^	26	Belgium	0.0	19.0	7.4	7.0†
Gao et al.^16^	54	China	0.0	0.0	7.4	13.5
Menegozzo et al.^17^	31	Brazil	0.0	26.0	0.0	5.0
Mansour et al.^18^	30	Egypt	0.0	67.0	26.7	4.7
Current study	60	South Africa	0.0	28.3	23.3	4.0‡

Note: Please see the full reference list of the article, Mohlala L, Moeng MS, Nweke EE, Makhadi S. Laparoscopy in abdominal trauma at a high-volume Level 1 trauma centre in a low- and middle-income setting: A 54-month retrospective analysis. J Coll Med S Afr. 2026;4(1), a494. https://doi.org/10.4102/jcmsa.v4i1.494, for more information.

LOS, length of stay; US, United States; IQR, interquartile range.

† , Median;

‡ , Median (IQR 3–7 days).

## Discussion

This 54-month retrospective analysis demonstrates that laparoscopy is feasible with acceptable observed short-term outcomes for the management of abdominal trauma in a resource-constrained, high-volume Level 1 trauma centre in South Africa. With no in-hospital mortalities, a complication rate of 6.7% (95% CI: 2.6% – 15.9%), and a negative laparoscopy rate of 28.3% (95% CI: 18.5% – 40.8%), our findings add to the growing body of evidence supporting the investigation of laparoscopy in trauma, even in settings far removed from the well-resourced centres where the technique was first developed. The absence of a comparator group precludes conclusions about comparative effectiveness; outcomes are reported descriptively for the selected cohort.

### Laparoscopy as a gatekeeper against unnecessary laparotomy

A key finding in our cohort was that 17 of 60 patients (28.3%, 95% CI: 18.5% – 40.8%) had negative laparoscopy, suggesting that laparoscopy may have avoided non-therapeutic laparotomy in over a quarter of patients, a procedure associated in the literature with a complication rate of up to 14.5%, a 33% increased adjusted mortality risk and significant healthcare resource utilisation.^6,7^ This rate is consistent with the 25% – 37% negative laparoscopy rates reported by Awad et al.,^14^ Menegozzo et al.,^17^ and Koto et al.,^13^ and higher than the 16.7% in our institution’s previous series.^12^ It should be noted, however, that without a comparative laparotomy cohort, the actual benefit in terms of avoided complications cannot be directly quantified from this study.

The negative laparoscopy rate increased from 16.7% in our previous series^12^ to 28.3% in the current cohort. The reason for this change is not clear from this retrospective dataset, as data on selection criteria or their evolution over time were not systematically collected. Potential explanations include broadening of diagnostic indications or changes in case mix, but these remain speculative. In resource-limited settings where hospital beds are at a premium and theatre access is competitive, characterising the rate of negative laparoscopy is important for ongoing quality assessment of patient selection practices.

### The unique context of trauma laparoscopy in low- and middle-income countries

The application of laparoscopy in LMICs faces a fundamentally different landscape from that in high-income settings. South Africa contends with a quadruple burden of disease, human immunodeficiency virus (HIV) or tuberculosis, non-communicable diseases, maternal and child health challenges, and injuries and violence,^1^ all competing for the same limited resources. Trauma rates in this context are five to nine times the global average, and the surgical workforce density remains critically insufficient.^3,19^ Systematic reviews of laparoscopy in LMICs have consistently identified barriers including equipment costs, lack of trained personnel, unreliable infrastructure and absence of standardised training pathways.^10,11^

Our study demonstrates that despite these challenges, sustained laparoscopic practice in trauma is achievable. Over a 54-month period, laparoscopy was performed in 60 trauma patients with short-term outcomes, including zero in-hospital mortality, a 6.7% complication rate and a 4-day median length of stay, that are comparable to published series from better-resourced settings. These descriptive findings are encouraging but must be interpreted with caution given the absence of a control group and the selected nature of the cohort.

Resource constraints in LMIC settings have been proposed in the literature as creating a potential imperative for minimally invasive approaches, given that shorter operative times, reduced postoperative complications and earlier discharges, where demonstrated, would represent proportionally greater benefits in settings where resources are more limited.^10^ These potential advantages were not directly measured in the current study, which lacked a comparator laparotomy group. Nonetheless, the median length of stay of 4 days compares favourably with open surgery series in similar contexts, warranting prospective evaluation. Access to emergency theatre remains a challenge at our institution, with some patients awaiting the weekly elective slate for procedures such as suspected diaphragmatic injury repair, creating cascading delays for other cases.

### Conversion rates and the training imperative

Our conversion rate of 23.3% (95% CI: 14.4% – 35.4%) is towards the higher end of published series (range 0% – 26.7%^18^), though it must be contextualised. The majority of conversions were for definitive repair (15.0%), representing cases where laparoscopy successfully identified the injury, but open surgery was required to complete the repair, rather than failure of the laparoscopic approach per se. Only one conversion (1.7%) was for poor visualisation, suggesting that the diagnostic utility of laparoscopy was largely preserved even when therapeutic completion required conversion.

The conversion rate in our previous institutional series was 14.8%,^12^ and the increase in the current cohort (23.3%) warrants investigation. Surgeon experience was not recorded as a variable in this retrospective dataset, precluding conclusions about the relationship between operator experience and conversion. This represents a limitation and should be addressed in future prospective studies. Regardless of cause, the finding highlights a need for structured, competency-based laparoscopic training programmes.^10,11,20^ In sub-Saharan Africa, the scarcity of experienced laparoscopic mentors, limited access to simulation facilities and the high-pressure clinical environment create significant obstacles to skill acquisition.^11^ Our data suggest that while laparoscopy can be safely introduced and sustained, continued investment in training infrastructure is essential to optimise conversion rates and expand the scope of injuries managed laparoscopically.

### Patient selection and clinical decision-making

All patients in this cohort were haemodynamically stable, consistent with the established prerequisite for laparoscopy in trauma.^5,8,9,14,15,16,17^ The admission clinical parameters (median Systolic Blood Pressure [SBP] 129.5 mmHg, pH 7.366, base excess −3.55, lactate 2.1 mmol/L) are consistent with the selection criteria applied. Computed tomography findings, where available, guided operative planning and identified findings such as pneumoperitoneum, free fluid and diaphragmatic injuries.

The lack of statistically significant correlations between admission physiological parameters and length of stay on Spearman analysis (Table 4) likely reflects the homogeneity of this selected cohort; all patients were stable enough to undergo laparoscopy, resulting in a narrow range of physiological derangement. This finding should not be interpreted as evidence that these parameters are unimportant in-patient selection; rather, it suggests that the selection process effectively identified patients for whom laparoscopy was appropriate, resulting in uniformly favourable outcomes.

The absence of significant correlations between admission physiological parameters and length of stay likely reflects the homogeneity of this selected cohort; all patients were stable enough to undergo laparoscopy, resulting in a narrow physiological range. Future prospective studies should include a comparator group and collect surgeon experience data to better characterise determinants of outcomes in this population.

### Building on the institutional evidence base

This study represents the continuation of a programmatic commitment to laparoscopy in trauma at our institution. Our previous series of 54 patients^12^ demonstrated the feasibility of introducing laparoscopy; the current study of 60 patients demonstrates its sustainability. The similarity in cohort sizes across these two time periods, both approximately 50–60 patients, provides insight into the practical throughput of trauma laparoscopy at a single centre with the resource constraints typical of LMIC public hospitals.

Comparing the two series, several descriptive trends are noted. The negative laparoscopy rate increased from 16.7% to 28.3%. The conversion rate increased from 14.8% to 23.3%; the reason for this increase is unclear from the available retrospective data, as surgeon experience was not recorded in either series. The complication rate remained low (6.7% in the current series), and both series recorded zero in-hospital mortalities. These findings collectively support the continued feasibility of the approach while highlighting areas, particularly conversion optimisation and training, warranting further investigation.

### Context within the global literature

Our findings are broadly consistent with the international literature on laparoscopy in trauma (Table 5). The zero in-hospital mortality in our series aligns with the majority of published studies,^5,12,14,15,16,17,18^ with the exception of Koto et al.^13^ (11.4%, in a predominantly blunt trauma cohort) and Gao et al.^16^ (1.9%). The length of stay in our cohort (median 4 days) compares favourably with most series, and is substantially shorter than the 11 days reported by Koto et al.^13^ and 13.5 days by Gao et al.^16^

Notably, our study is one of the few to specifically address the LMIC context. While the majority of the evidence base comes from single-centre studies with modest sample sizes, the emerging picture from centres in South Africa,^12,13^ Egypt,^14,18^ Brazil^17^ and Colombia^5^ demonstrates that the benefits of laparoscopy in trauma are not confined to well-resourced settings. The challenge lies in scaling these practices beyond individual centres of excellence to achieve broader adoption across the LMIC surgical landscape.

### Limitations

This study has several limitations inherent to its retrospective design. Firstly, incomplete medical records resulted in the exclusion of nine patients for incomplete data. Secondly, as a single-centre retrospective study without a comparator group (laparotomy cohort), the findings can describe outcomes in the selected cohort but cannot establish comparative safety, effectiveness or resource savings relative to open surgery. Thirdly, surgeon experience was not recorded as a variable, precluding analysis of its relationship with conversion rates or outcomes. Fourthly, the relatively small sample size limits statistical power. Fifthly, long-term outcomes including delayed complications, quality of life, and return to function were not assessed. Sixthly, all claims regarding resource savings, reduced morbidity and shorter operative times are based on external literature rather than direct comparison within this dataset and should be interpreted accordingly.

## Conclusion

This 54-month retrospective analysis demonstrates that laparoscopy is feasible with acceptable observed short-term outcomes for selected haemodynamically stable patients with abdominal trauma (predominantly penetrating), even in the challenging environment of a resource-constrained, high-volume Level 1 trauma centre in South Africa. Laparoscopy potentially avoided non-therapeutic laparotomy in 28.3% of patients, achieved definitive repair in 31.7% without conversion and was associated with zero in-hospital mortality, a low complication rate (6.7%, 95% CI: 2.6% – 15.9%), and a median hospital stay of 4 days (IQR 3–7). These outcomes are reported for a highly selected cohort and should not be extrapolated as evidence of comparative superiority over open surgery.

These findings add to the growing body of descriptive evidence supporting the feasibility of laparoscopy in LMIC trauma settings. The conversion rate of 23.3% underscores the need for structured investment in laparoscopic training programmes, particularly in sub-Saharan Africa where trainees face limited access to structured minimally invasive surgical training.

Prospective multicentre studies comparing laparoscopy with laparotomy in LMIC trauma settings are needed to establish evidence of comparative effectiveness, develop validated patient selection tools, and inform resource allocation for laparoscopic infrastructure. Future studies should include a total trauma denominator, record surgeon experience and assess long-term outcomes.
